# Docosahexaenoic acid increases accumulation of adipocyte triacylglycerol through up-regulation of lipogenic gene expression in pigs

**DOI:** 10.1186/s12944-017-0428-3

**Published:** 2017-02-07

**Authors:** Chao-Wei Huang, Yu-Jen Chen, Jui-Ting Yang, Ching-Yi Chen, Kolapo M. Ajuwon, Shuen-Ei Chen, Nan-Wei Su, Yu-Shan Chen, Harry J. Mersmann, Shih-Torng Ding

**Affiliations:** 1 Department of Animal Science and Technology, National Taiwan University, No.50, Ln. 155, Sec. 3, Keelung Rd., Da’an Dist, Taipei City, 10672 Taiwan; 2 Institute of Biotechnology, National Taiwan University, Taipei, 10672 Taiwan; 3 Department of Animal Science, National Chung-Hsing University, Taichung, 40227 Taiwan; 4 Department of Animal Sciences, Purdue University, West Lafayette, IN 47907-2054 USA; 5 Department of Agricultural Chemistry, National Taiwan University, Taipei, 10672 Taiwan

**Keywords:** Docosahexaenoic acid, Lipid metabolism, Protein kinase A, Adipose tissue

## Abstract

**Background:**

Changing dietary fatty acid composition in modern diet influences the prevalence of obesity. Increasing evidences suggest favorable effects of n-3 PUFA for protecting against obesity and the metabolic syndrome. However, the regulation of n-3 PUFA in adipose is still unclear. Thus, this study addressed metabolism of different dietary fats in the adipose tissue of porcine model.

**Methods:**

Eight-week-old cross-bred pigs were randomly assigned to three groups and fed a 2% fat diet for 30 days from either soybean oil (SBO), docosahexaenoic acid (DHA) or beef tallow. An in vitro experiment was conducted in which linoleic acid (LA), DHA or oleic acid (OA) were added to represent the major fatty acid in the SBO-, DHA- or BT- diets, respectively. Adipocytes size and lipid metabolism related genes were analyzed.

**Results:**

Plasma triacylglycerol (TAG) was lower in DHA- than in BT-fed pigs, and the product of lipolysis, glycerol was highest in BT-fed pigs. In addition, expression of the lipolytic genes, adipose triglyceride lipase and hormone sensitive lipase was higher in BT-fed pigs and with OA treatment in vitro. DHA promoted protein kinase A activity in pigs without affecting lipolytic genes. Adipocyte cell sizes, TAG content and expression of lipogenic-related genes including, adipose differentiated related protein (ADRP) and diacylglycerol acyltransferase 1 (DGAT1) were elevated by DHA in vivo and in vitro, indicating DHA promoted adipogenesis to trap TAG in adipose tissue. Fatty acid β-oxidation genes were increased in the DHA-fed pigs.

**Conclusion:**

This effect was partly explained by the effect of DHA to promote adipogenesis to trap TAG in adipocytes and also increase expression of genes involved in adipocyte fatty acid oxidation. Therefore, our results suggest a direct effect of DHA on adipocyte metabolism, resulting in TAG turnover and fatty acid dissipation to facilitate plasma lipid uptake from the circulation.

**Electronic supplementary material:**

The online version of this article (doi:10.1186/s12944-017-0428-3) contains supplementary material, which is available to authorized users.

## Background

In humans, dietary fats are very important for energy homeostasis because they contributes to approximately 30% of the daily energy intake [1]. Dietary fatty acid (FA) composition is an important factor in weight control because the intake of saturated fatty acids is linked to increased risk for obesity [2–4]. In contrast, dietary polyunsaturated fatty acids (PUFA) suppress the metabolic syndrome phenotype by lowering plasma triacylglycerols (TAGs). Markers of the metabolic syndrome, including dyslipidemia, hyper-insulinemia, and hepatic steatosis are attenuated by PUFA [5–8]. The serum TAG lowering by the n-3 series of fatty acids is demonstrated in obese children compared to age-matched lean controls [9]. Increased consumption of PUFA is associated with reduced weight gain and an improved metabolic profile [10]. In addition, diets with a lower ratio of n-3 to n-6 fatty acids may lead to the pathology of metabolic syndrome in children [11], and consumption of a low dose of n-3 PUFA (1 g of n-3 PUFA/day) is associated with a reduction in plasma TAG in humans [12]. Therefore, although the benefits of n-3 PUFA have been well documented, the underlying mechanisms involving adipocytes have not been clearly elucidated.

Pigs have substantial amounts of adipose tissues and adipose tissue is the main site for de novo fatty acid synthesis [13]. Subcutaneous adipose tissues (SCAT) also play a more active role in the regulation of whole-body metabolism than visceral fat, which is associated with metabolic process but not the metabolic risk factor as visceral fat [14]. Therefore, adipose tissue of the pig is an excellent platform for elucidating the direct effects of fatty acids on subcutaneous adipose tissue and its effects on whole body metabolism. Even though de novo fatty acid synthesis occurs primarily in pig adipose tissue, porcine back fat transcriptome analysis indicates expression of several genes involved in lipid metabolism is similar to human [15]. Increasing the n-3: n-6 ratio exerts beneficial effects on lipid metabolism and inflammatory systems in the pig [16]. Pigs (28 days of age) fed with 10% DHA algal oil or soybean oil for 2 days have similar mRNA expression of sterol regulatory element binding protein (SREBP-1), a transcription factor associated with lipogenesis and expression of acyl-coenzymes A oxidase 1 (ACOX1), an enzyme involved in fatty acid oxidation in adipose tissue [17]. Similarly, a study by Hsu et al., 2004 shows that 30-day-old crossbred pigs fed for 18 days with either 2% tallow or DHA oil have the same body weight with no differences in adipose tissue SREBP-1 expression [18]. Even though the SREBP-1 expression is not affected in adipose tissue, serum TAG is lower in both studies after DHA treatment. Furthermore, in pigs fed a diet with 330 mg (low), 3600 mg (medium) or 9400 mg (high) DHA per day for 28 days before slaughter at market weight (~110 kg), the high dose of DHA promotes ACOX1 and peroxisome proliferator-activated receptors alpha (PPARα) and gamma (PPARγ) expression in the adipose tissue [19]. The 9400 mg DHA group also has decreased plasma TAG. These results indicate that lowering plasma TAG by DHA is partially mediated by pig SCAT via several mechanisms. However, based on the in vivo studies the TAG lowering effect of PUFA might be regulated by other organs as well.

In this study, we hypothesized that the benefits of n-3 PUFA, particularly DHA, in lowering plasma TAG, involves a direct effect on adipocyte lipid metabolism. To establish the effect of different fatty acids in adipocytes, pigs were fed diets with various fats and porcine adipocytes in vitro were treated with the major fatty acids mimicking the treatment diets.

## Methods

### Animals

All animal experiments described were approved by the Animal Care and Use Committee of the National Taiwan University (IACUC approval NO: NTU-102-EL-3). Thirty cross-bred, Landrace × Yorkshire × Duroc pigs were purchased from a commercial pig farm and housed at the experimental farm of NTU. They were randomly assigned to one of three dietary groups each with five castrated males and five females. Animals were allowed to adapt for 7 days to a control diet after assignment (National Research Council 1998) and the body weight, in each group was not significantly different after the adaption period in each group (18.80 ± 2.22 kg, 18.25 ± 2.37 kg, 18.20 ± 1.25 kg, respectively). The control diet contained 21.33% crude protein, 3.22% crude fiber, 2.17% fat, 0.75% calcium, 0.68% phosphorus, 0.21% sodium, 29.12 mg kg^−1^ zinc, 12.25 mg kg^−1^ copper in a pelleted feed consisting of 35% corn, 25% peas, 19% barley, 17% canola, and vitamin premix including 13.25 mg kg^−1^ vitamin E and 0.55 mg kg^−1^ selenium. The pigs were then fed the experimental diets (Table 1) supplemented with 2% (as-fed basis) with beef tallow (BT), soybean oil (SBO) or DHA oil (DHASCO, Martek Biosciences Corp., Columbia, MD, USA) for 30 days. The fatty acid compositions of the oils and diets are indicated in Additional file 1: Table S1 and S2, respectively. Feed and water were provided ad libitum throughout the duration of the study.

**Table 1:** Compositions of experimental diets (wt/wt, as-fed basis)

Diet^a^	SBO (%)	DHA (%)	BT (%)
Corn	54.9	54.9	54.9
Soybean meal, solvent extracted	30.15	30.15	30.15
Skimmed milk	10	10	10
Soybean oil	2	–	–
DHA oil^b^	–	2	–
Beef tallow	–	–	2
NaCl iodide	0.35	0.35	0.35
CaCO3	0.9	0.9	0.9
CaHPO4	1.2	1.2	1.2
Vitamin premix	0.25	0.25	0.25
Mineral premix	0.25	0.25	0.25
Total	100	100	100
Crude protein	20.9	20.9	20.9
Crude fat	4.08	4.08	4.08
Calcium	0.74	0.74	0.74
Phosphorus	0.67	0.67	0.67
Metabolizable energy (ME, kcal/kg)^c^	3271	3342	3265

^a^Diets were different oils. *SBO* soybean oil, *DHA* docosahexaenoic acid oil, *BT* beef tallow

^b^DHA oils extracted from algae containing about 44% of DHA

^c^Metabolizable energy in SBO-, DHA- and Beef-tallow diet are: 3271 kcal/kg, 3342 kcal/kg and 3265 kcal/kg, respectively

### Sample collection and preparation

Pigs were weighed before the start of the experiment and once every week thereafter. At week 4, blood samples were collected from the anterior vena cava using EDTA as anticoagulant after a 12 h overnight fasting. After 30 days of feeding, pigs were sacrificed by electrical stunning coupled with exsanguination. SCAT from the dorsal neck region, including both the upper and middle layers and liver, were snap-frozen in liquid nitrogen and stored at −80 °C prior to processing. Plasma was separated by centrifugation (2000 × g for 10 min at 4 °C) and stored at −80 °C.

### Measurement of triacylglycerol, free fatty acid and glycerol

Plasma of TAG, FFA, glycerol and TAG in tissues, such as liver and adipose tissue were measured in duplicate using commercially enzyme-based kits according to the manufacturer’s instructions (Cayman Laboratories, Ann Arbor, Michigan, USA); TAG (10,010,303), glycerol (10,010,755) and free fatty acids (700,310).

### RNA extraction and gene expression analysis by quantitative real-time polymerase chain reaction (qRT-PCR)

Total RNA was extracted from tissues and cells using TRIzol (Invitrogen, Carlsbad, CA, USA). Genomic DNA was then removed from the RNA samples using the TURBO-DNase free kit (Applied Biosystems, Foster City, CA, USA) followed by reverse transcription into cDNA using the High Capacity cDNA Reverse Transcription kit (Applied Biosystems). The qRT-PCR, reactions were performed with the RealQ-PCR Master Mix kit (Ampliqon, Herlev, Denmark) on a LightCycler 480 Instrument II (Roche Diagnostics, Indianapolis, IN, USA). Running conditions for real-time PCR were; initial denaturation at 95 °C for 7 mins followed by 39 cycles of denaturation at 95 °C for 10 s, followed by annealing/extension at 60 °C for 30s and a terminal extension at 60 °C for 1 min. A melt curve was generated with a temperature gradient from 60 to 95 °C in increments of 0.5 °C, each for 5 s. Primers used for amplification are listed in Table 2. The threshold cycle (Ct) values were obtained, and the relative gene expression was calculated using the comparative Ct method [20]. The relative value of each target gene was normalized to β-actin expression in the same sample. All samples were analyzed in triplicate, and the PCR amplification efficiency was close to 100%. Amplification of specific transcripts was further confirmed by melting-curve analysis and agarose-gel electrophoresis.

**Table 2:** List of primer sets for quantitative PCR by the SYBR system

Gene Name	NCBI accession number	Forward primer (5’-3’)	Reverse primer (5’-3’)	Product size
ap2	NM_001002817.1	TGGTACAGGTGCAGAAGTGG	TTCTGGTAGCCGTGACACCT	108
ACOX1	NM_001101028.1	TTGGCCCCAAATTCGGCTAT	GGCTTCACCTGGGCATACTT	106
ADRP	NM_214200.2	GCTGGTGAGCAGTGGAGTAG	ACTTGGCTTCTGAACCATATCA	136
ATGL	NM_001098605.1	TTCCCCAAAGAGACGACGTG	CGGTGATGGTGCTCTTGAGT	196
β-actin^a^	AY550069	GCCAGGTCATCACCATCGG	GTAGAGGTCCTTGCGGATGTC	103
CD36	NM_001044622.1	TGGGTTAAAACAGGCACGGA	TGCTACTTCCTCTGGATTCTGC	123
CGI-58	NM_001012407.1	TTTTCCTGAGCGACCAGACC	GTCCTGCTCCAAGAATGGCT	163
CPT1α	NM_001129805.1	AAGGACATGGGGAAGTTTTC	ACGTAGAGGCAGAACAGGTG	247
DGAT1	NM_214051.1	TTCTACAAGCCCATGCTCCG	AGAAGGCTGAAGCCAGGAAC	80
FAS	NM_001099930.1	GTGGGCTACAGCATGATAGG	GAATTGCAGCGAGGAGTTAG	189
HSL	NM_214315.1	CCCTCGGCTGTCAACTTCTT	GGTGCTAATCTCGTCTCGGG	102
LPL	NM_214286.1	TGGACGGTGACAGGAATGTA	AAGGCTGTATCCCAGGAGGT	72
MGL	NM_001143718.1	ACTTCTCCGGCATGGTTCTG	GGGACATGTTTGGCAGGACA	114
Perilipin	NM_001038638.1	CAACAAGGGCCTGACTTTGC	GTTGGCGGCATATTCAGCAG	190
PPARα	NM_001044526.1	CAGC CTC CAGC C CCTCGTC	GCGGTCTCGGCATCTTCTAGG	381
PPARγ	NM_214379.1	TCGCATCTTTCAGGGGTGTC	CCCAGGGATGTTCTTGGCAT	79
SREBP1	AY338729.1	CCACCAGTCCTGATGCCAG	GTACATCTTCAGCGGGGTGG	152

*Abbreviations*: *ap2* adipocyte protein 2, *ACOX1* acyl CoA oxidase, *ADRP* adipose differentiation related protein, *ATGL* adipose triglyceride lipase, *CGI58* comparative gene identified-58, *CPT1α* carnitine palmitoyltransferase 1α, *DGAT1* diglyceride acyltransferase 1, *FAS* fatty acid syntheses, *HSL* hormone sensitive lipase, *LPL* lipoprotein lipase, *MGL* monoglyceride lipase, *PPARα* peroxisome proliferator-activated receptor α, *PPARγ* peroxisome proliferator-activated receptor γ, *SREBP1* sterol response element binding protein

^a^reference gene

### Adipose tissue histology

SCAT was rapidly removed from the animals, fixed in 10% formalin overnight and embedded in paraffin. Tissue blocks were sectioned at 5 μm thickness and stained with hematoxylin-eosin (H&E). Stained slides were observed and photographed. The regions of tissue slices analyzed were chosen randomly from three areas in each tissue. The cellular diameter within the SCAT was calculated using the image-analysis program, Image J (NIH, Bethesda, MD, USA) [21]. In brief, 200 random adipocytes from a representative photomicrograph from each pig (*n* = 10 pigs per group) were analyzed for cell size using the program.

### PKA activity of SCAT

The activity of PKA in pig subcutaneous fat previously stored at −80 °C was measured using the PepTag assay for nonradioactive detection of cAMP-dependent PKA following the manufacturer’s instructions (Promega, Madison, WI, USA).

### Isolation of porcine stromal/vascular cells and differentiation of porcine adipocytes

The cross-bred suckling (7 ~ 9 days from birth) Landrace × Yorkshire × Duroc animals with the same genetic background as for the study in-vivo were purchased from a commercial farm. The protocol for stromal/vascular (S/V) cell isolation was modified from Chen et al. [22]. In brief, adipose samples were removed from the dorsal neck and back SCAT. The tissue mass was minced with scissors and digested with type I collagenase (Sigma-Aldrich Chemical, St. Louis, MO, USA) for 90 min in a 37 °C shaking water bath. The isolated S/V cells were centrifuged at 700 × g for 10 min to separate them from the floating adipocytes. The S/V cells were seeded on culture plates at a density of 1 × 10^6^ cell/cm^2^. Cells were cultured to confluence for 2 days in DMEM/F12 (Thermo Fisher Scientific, Lafayette, Colorado, USA) culture medium containing 10% fetal bovine serum (FBS) and 1% antibiotics (penicillin-streptomycin-amphotericin B solution). Then the medium was replaced with serum-free, hormone supplemented differentiation medium (DMEM/F12 containing sodium bicarbonate, 0.5 μM insulin, 10 mg/L transferrin, 100 nM dexamethasone, 1 μM rosiglitazone, 100 kU/L penicillin, 100 mg/L streptomycin and 1.5 mg/L amphotericin B) for 12 days to induce adipogenesis. The medium was replaced every 3 days. On day 12, the well-differentiated adipocytes were treated for 48 h with a serum-free DMEM/F12 medium containing 100 μM of linoleic acid (LA; 90,150), DHA (90,310) (Cayman Laboratories, Ann Arbor, MI, USA) or oleic acid (OA; O1008, Sigma-Aldrich Chemical, St. Louis, MO, USA) bound to 1% fatty acid free, bovine serum albumin (US Biological, San Antonio, TX, USA). The final albumin concentration was 1% in all culture media, including the control with no exogenous FAs. The selection of exogenous FAs for the study in vitro was used to test the effect of the major FAs in the plasma (saturated, monounsaturated, n-3 PUFA and n-6 PUFA (Additional file 1: Table S3)) of the animals. These major FAs were LA, DHA and OA, which represent n-6, n-3 and monounsaturated fatty acids, respectively). Effects of different fatty acids on the adipocyte phenotype and gene expressions were determined as described previously for adipose tissue [23]. Media were collected from all treatments and assayed for glycerol and FFA concentrations using methods described above. Results were expressed as means ± S.E.M. for 6 independent culture experiments using S/V cells isolated from 6 different pigs.

### Statistical analysis

Given there were no sex effects (*P* >0.05), data were analyzed using one-way analysis of variance (ANOVA). Tukey’s test was used to determine differences between means (SAS institute, Cary, NC, USA). Data are expressed as means ± SEM. Additional file 1 of fatty acid compositions are expressed as means ± SD. *P* values ≤ 0.05 were considered statistically significant. If the fatty acid is non-detectable in one of the groups, pairwise comparison of two groups (fatty acids analysis; Additional file 1: Table S1, S2, S3 and S4) was done with the Student’s *t* test.

## Results

### Weight gain and plasma metabolites

**Fig. 1:**
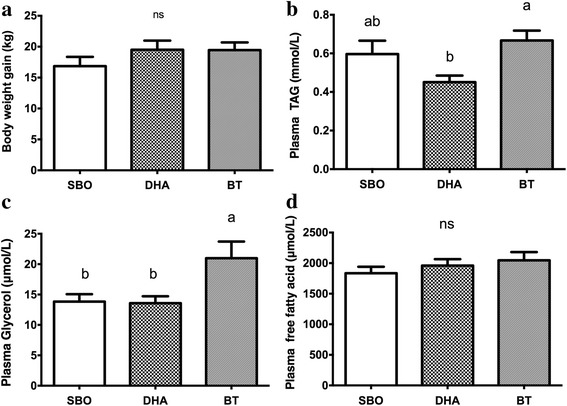
Body weight gain during experiment period **a**. Plasma analysis, plasma triacylglycerol (TAG, **b**), glycerol **c** and free fatty acids (FFA, **d**) after dietary treatment with 2% dietary soybean oil (SB), docosahexaenoic acid (DHA) or beef tallow (BT) for 30 days. There were 10 pigs per dietary group. Data are means ± S.E.M. One-way ANOVA followed by Tukey’s post hoc test was performed for multiple comparisons. Bars with different letters above represent statistical significance at *p* ≤ 0.05. ns represent no significant difference between each group

The body weight gains were 16.85 ± 4.76 kg in the SBO-fed pigs, 19.50 ± 4.68 kg in the DHA group and 19.45 ± 3.93 kg in BT-fed pigs (Fig. 1a), and these were not different between treatments. In addition, the average feed intakes between the groups were not different (total 30 days feed intake were 0.870 kg/day, 0.867 kg/day, and 0.866 kg/day for the SBO-, DHA- and BT-fed pigs, respectively). DHA concentrations in the plasma and adipose tissues were increased in the DHA-fed group, indicating that the dietary DHA oil can be utilized to increase tissue DHA deposition in pigs (Additional file 1: Table S3 and S4). Plasma TAG was lower in the DHA-fed compared to the BT-fed group (Fig. 1b). Of the two indicators of lipolysis, plasma glycerol but not FFA, was elevated in the BT-fed pigs compared to the DHA- or SBO-fed pigs (Fig. 1c and d).

### Adipose size and tissue TAG content

**Fig. 2:**
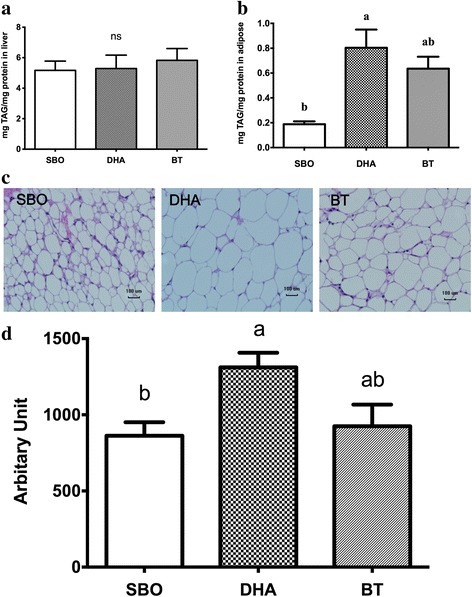
Tissues analysis. TAG in liver **a** and adipose tissue **b** Histological adipocyte areas in subcutaneous fat using H&E staining **c** Quantitative representation of average adipocyte area in subcutaneous fat in a 100-μm^2^ area **d** Quantification of the size of adipocytes was by Image J analysis. All conditions were as in Fig. 1. Adipocyte area was determined from 10 pigs/group. ns represent no significant difference between each group

DHA-fed pigs had increased adipocyte size compared to SBO-fed pigs (Fig. 2c and d). This result is consistent with the adipose tissue TAG concentrations obtained from these pigs (Fig. 2b). The liver TAG contents were not affected by the different dietary fats (Fig. 2a).

### Individual dietary fats differentially affect gene expression in subcutaneous adipose tissue

**Fig. 3:**
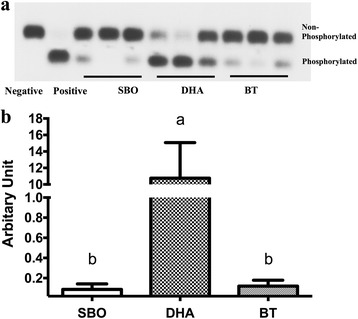
Protein kinase A phosphorylation in adipose tissue. **a** PKA phosphorylation activity **b** quantification of the ratio of phosphorylated to non-phosphorylated PKA. All conditions were as in Fig. 1 (*n* = 10 pigs/group)

Protein kinase A (PKA) is an important kinase that controls many enzymes in the lipolytic pathway including hormone sensitive lipase (HSL). In the DHA-fed group, PKA phosphorylation was greatly increased compared to the BT- or SBO-fed groups (Fig. 3a and b).

**Fig. 4:**
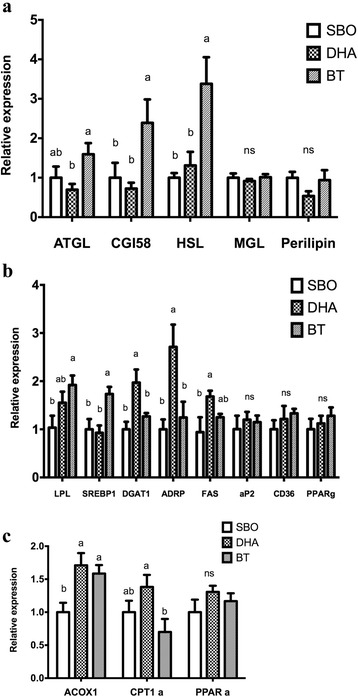
Lipolysis- **a**, lipogenesis- **b** and fatty acid oxidation-related **c** gene expression in adipose tissue. Lipolysis genes = lipoprotein lipase (LPL), adipose triglyceride lipase (ATGL), comparative gene identified 58 (CGI58), hormone sensitive lipase (HSL) and monoglyceride lipase (MGL). Lipogenesis-related genes = cluster of differentiation 36 (CD36), sterol regulatory element-binding transcription factor 1 (SREBP1c), diacylglycerol acyltrasnferase (DGAT1), peroxisome proliferator-activated receptor γ (PPAR γ), adipose differentiation related protein (ADRP) and fatty acid synthase (FAS). Fatty acid oxidation genes = peroxisome proliferator activated receptor α (PPAR*α*), acyl-CoA oxidase 1 (ACOX1) and carnitine palmitoyltransferase 1 (CPT1*α*). All conditions are as in Fig. 1 (*n* = 10 pigs/ group). ns represent no significant difference between each group

Expression of the lipolytic genes, ATGL, CGI58 and HSL was up-regulated by dietary BT (Fig. 4a). Lipoprotein lipase mRNA was greater in the BT-fed group than in the SBO-fed group (Fig. 4b). Expression of mRNA for SREBP-1 was greatest in the BT-fed pigs, intermediate in the SBO-fed pigs and lowest in the DHA-fed pigs (Fig. 4b). Compared with the other dietary groups, DHA treatment led to up-regulation of expression of the lipogenesis-related genes, DGAT and ADRP (Fig. 4b). Expression of PPARα, a transcription factor associated with fatty acid oxidation, was not significantly different between the groups (Fig. 4c). In the DHA-fed group, expression of the fatty acid oxidation gene, acyl CoA oxidase (ACOX1) was increased compared with the SBO-fed group and carnitine palmitoyltransferase 1α (CPT1*α*) was increased in the DHA-fed group compared with the BT-fed group (Fig. 4c). The results indicated that DHA treatment not only promoted expression of lipogenesis-related genes but also promoted the expression of fatty acid oxidation-related genes in subcutaneous adipose tissue.

### Dietary fatty acids affect the phenotype and gene expressions in porcine adipocytes in vitro

**Fig. 5:**
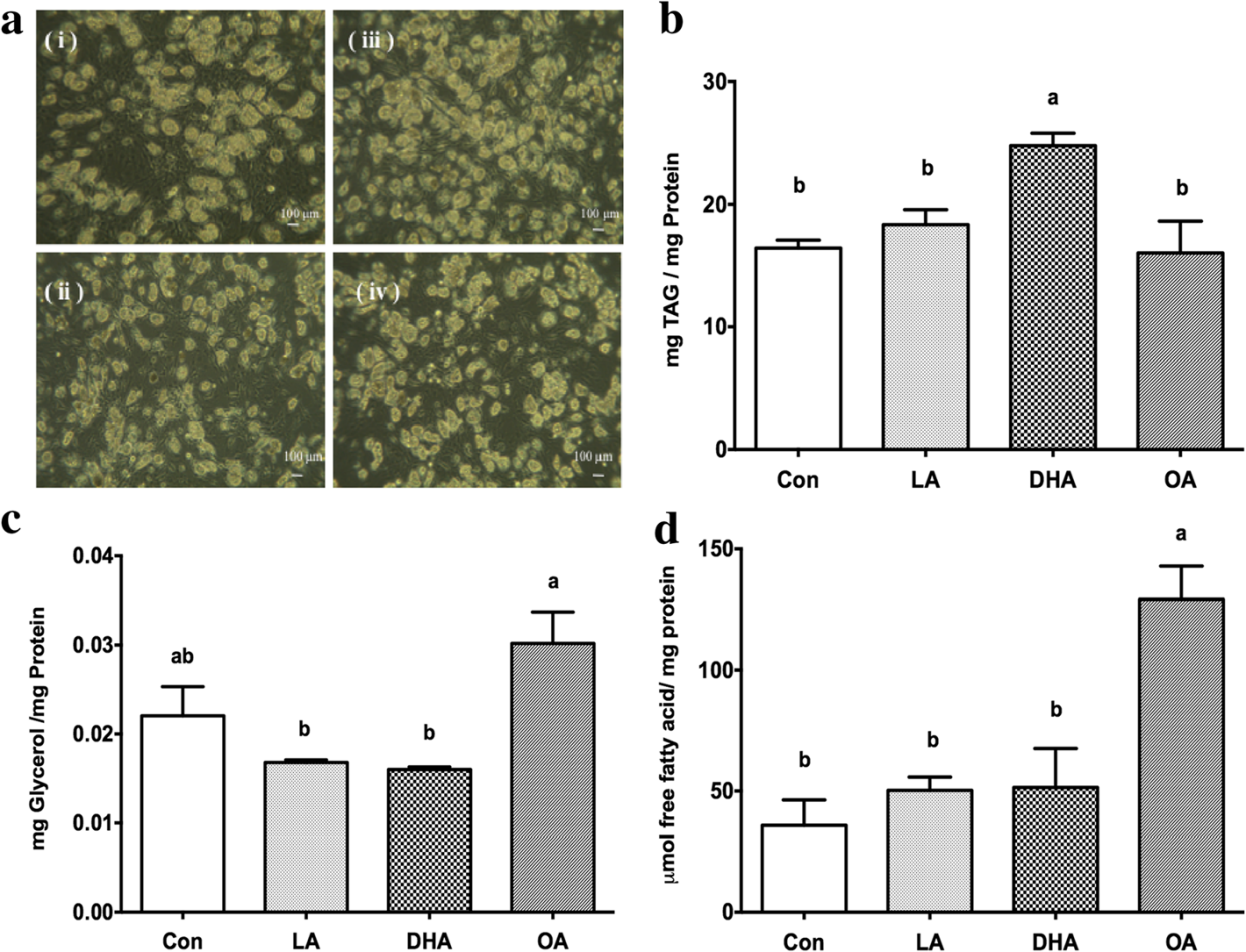
Analysis of adipocytes morphology and physiological condition in vitro. Porcine S/V cells were differentiated for 12 days then adipocytes were incubated with 100 μM fatty acids for 48 h. Morphology **a** and TAG content **b** in differentiated adipocytes in vitro. Medium glycerol **c** and FFA **d** released from the cells. The final albumin concentration was 1% in all culture media (i) Con (ii) LA (iii) DHA (iv) OA (Con = control, no exogenous FA added, LA = linoleic acid (C18:2n-6), DHA = docosahexaenoic acid (C22:6n-3) and OA = oleic acid (C18:1n-9). Images were taken at 200× magnification using phase contrast microscopy. Primary porcine adipocytes were incubated with 100 μM fatty acids for 48 h. *N* = 6 independent experiments, each with cells isolated from a different pig. Analysis of variance, followed by Tukey’s post-hoc test was used to determine statistic significances. *Bars* with different letters were statistically significant (*p* ≤ 0.05)

**Fig. 6:**
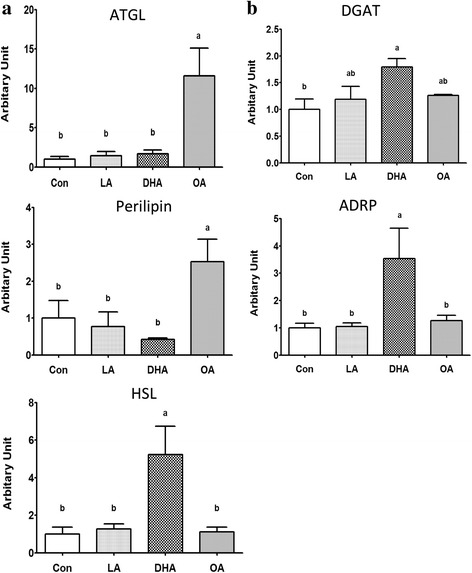
Lipolysis- **a** and lipogenesis **b**-related gene expression in pig differentiated adipocytes treated with or without 100 μM different fatty acids. The final albumin concentration was 1% in all culture media (i) Con (ii) LA (iii) DHA (iv) OA (Con = control, no exogenous FA added, LA = linoleic acid (C18:2n-6), DHA = docosahexaenoic acid (C22:6n-3) and OA = oleic acid (C18:1n-9). Lipolysis-related genes = adipose triglyceride lipase (ATGL), perilipin and hormone sensitive lipase (HSL). Lipogenesis-related genes = diacylglycerol acyltransferase (DGAT) and adipose differentiated related protein (ADRP) (*n* = 6). All conditions were as in Fig. 5

To elucidate the direct effect of dietary fatty acids on porcine adipocytes, adipocytes differentiated from S/V cells for 12 days were treated for 48 h with LA, DHA or OA to represent the major fatty acid composition in the diets of SBO-, DHA-, and BT-fed pigs, respectively. Thereafter, their phenotypes were observed (Fig. 5a) and cellular TAG content (Fig. 5b), glycerol and free fatty acid release to the medium (Fig. 5c and d, respectively) were measured. The TAG content of adipocytes was only increased by the DHA treatment (Fig. 5b). Indicators of lipolysis, glycerol and free fatty acids were increased in the medium by OA treatment (Fig. 5c and d) suggesting that OA promotes adipocyte lipolysis. Treatment with OA led to elevated ATGL (Fig. 6a). This is consistent with the in vivo data showing that the BT-fed group had an increase in ATGL (Fig. 4a). In conflict with the data in vivo, the expression of HSL was increased in DHA-treated cells (Fig. 6a). The lipogenic genes DGAT and ADRP were both elevated after DHA treatment, as observed in vivo (Fig. 6b).

## Discussion

The current study shows that consumption of DHA as the major fatty acid in the diet resulted in lower plasma TAG and increased expression of lipogenic genes in the SCAT. In humans, a daily intake of a low dose of fish oil, which provided n-3 PUFA (1 g/day) is associated with reduced plasma TAG [12, 24]. The dosage of PUFA equates to approximately 1.6% of the recommended daily dietary fat (60 g/d) based on Dietary Guidelines for Americans 2010. Our results agree with previous studies in pigs that indicate dietary supplementation with 2% n-3 PUFA is enough to reduce plasma cholesterol and TAG [17, 23, 25]. Despite the differences in the sites of de novo lipogenesis in humans (liver) and mice (considerable in the liver) versus pigs (adipose tissue), DHA exerts the same beneficial effects in lowering plasma TAG in mice, humans and pigs. We specifically focused on the effect of DHA supplementation during the growth phase of the pig, because understanding mechanisms during the growth phase may be important in developing nutritional strategies that optimize the utilization of DHA during growth.

Although measured in only a few animals via magnetic resonance imaging (MRI), total body fat was not different between dietary groups (data not shown). However, adipocyte size and tissue TAG levels were increased in the DHA-fed pigs and were intermediate in the BT-fed pigs compared to the SBO-fed pigs. These results suggest that consumption of DHA resulted in increased adipocyte TAG storage, leading to increased adipocyte size. The fact that DHA was detected in the circulation and was greatly elevated in adipose tissues of the DHA-fed group suggests that effects observed in adipose tissue in the DHA fed group may be directly linked to the dietary DHA (Additional file 1: Table S3 and S4). Dietary fish oil increases adipose tissue mass in ICR (imprinting control regions) mice, but is associated with improved insulin sensitivity and reduced hepatic steatosis [26, 27]. Sprague-Dawley rats fed a diet containing 30% fish oil for 6 weeks, have decreased epididymal fat pad weight compared to rats fed a mixture of vegetable and animal oil (mixture = 1.25 ± 0.08, fish oil = 0.97 ± 0.06; weight, g・100 g body), and symptoms of the metabolism syndrome are improved [28]. Overall effects of fish oil on adipose tissue may be independent of changes in adipose tissue mass and be partly accounted for by the genetic background of the subjects [29].

In a previous study, cross-bred pigs fed with beef tallow (BT) have greater proportions of SFA or MUFA, particularly oleic and stearic acids [30]. The current study indicated that BT-fed pigs had a higher proportion of all MUFA in the subcutaneous fat (Additional file 1: Table S4). In addition, when pigs are treated with a diet containing 10% of different dietary fat (BT group vs. high-oleic acid sunflower oil group, HOSO group) [31], the fatty acid content of oleic acid is higher in the BT group. However, stearoyl-CoA desaturease (SCD) is 40% lower in the adipose tissue of pigs fed HOSO. The results suggest that stearic acid in pigs is rapidly converted to oleic acid (18:1; OA) by the desaturase to a considerable extent [31]. Consequently, oleic acid was selected as our candidate FA to represent the BT diet in the in vitro study.

In a rodent models, increasing the OA content of the diet increases the lipolytic responses as characterized by glycerol and FFA [32]. Glycerol is positively correlated with the concentration of MUFA and negatively correlated with polyunsaturated fatty acids in the adipose tissue [33]. Furthermore, the lipolytic activity in epididymal adipose tissue and omental adipose tissue is dose-dependent on the dietary concentration of OA [34]. Consistent with our results, OA treatment of 3 T3-L1 adipocytes reduces lipid droplets compared with DHA treatment [35]. However, dietary BT supplementation also decreases lipolytic activities by reducing β-adrenergic receptor abundance in rats. Body weight is not affected, but the abdominal adipose tissues is increased by dietary BT treatment [36, 37]. It should be noted that these diet contained 20% of fat, which was about 4 times more than our diets (20% vs. 4.08%). When the whole body fat content was analyzed with EchoMRI, no difference among our dietary treatment groups was observed. The results indicated that although abundant BT in the diet may cause abdominal adipose tissue accumulation, it is unlikely to happen at low dietary fat content such as 4.08% in the current study. Our BT-fed pigs compared to the SBO- and DHA-fed pigs had increased lipolysis that was supported by similar effect in porcine adipocytes treated with OA in vitro. Oleic acid inhibits the lipogenic gene expression, including C/EBP*α* (CCAAT-enhancer binding proteins *α*), PPARγ and aP2 (adipocyte protein 2) compared with DHA treatment in 3 T3-L1 cells in vitro [35]. However, other studies indicate that OA increases the lipogenic gene expression [38]. The results suggest that OA may inhibit lipid accumulation after short-term treatment (48 h), but increase lipid storage after longer treatment time (5 days). Species and experimental design differences are suggested because differentiation of porcine S/V cells is stimulated by treatment with OA for 1, 5 or 10 days during adipocyte differentiation [38].

In pigs, DHA increases the secretion of serum amyloid A (SAA), which promotes lipolysis by the liver [39]. However, pig adipocytes treated with recombinant SAA protein have reduced HSL and ATGL expression [40]. The lower expression of HSL and ATGL in adipose tissue in our DHA-fed pigs may be associated with increased SAA expression [41, 42], although SAA concentration was not determined in this study. In our study in vitro, DHA treatment resulted in elevated HSL, but ATGL, an enzyme that is critical for the first reaction of lipolysis was not affected (Fig. 6a). The overall effect of DHA was a reduction in adipocyte and adipose tissue lipolysis that is associated with the reduced serum glycerol in DHA-fed pigs. Lipoprotein lipase (LPL) is the rate-limiting enzyme for the importation of plasma lipoprotein TAG-derived fatty acids into adipose tissue [43–45]. The LPL activity is implicated as a determinant of body composition, as well as development of obesity [46, 47]. Thus, increased LPL mRNA expression in the DHA-fed pig could partly account for the increased accumulation of TAG in adipocytes.

The activity of PKA promotes lipolysis through increased phosphorylation, activation of HSL and by phosphorylation and down-regulation of perilipin, allowing more enzymatic access to the TAG surface [48]. Increased PKA phosphorylation obtained in the adipose tissue from DHA-fed pigs was consistent with previous studies indicating that DHA promotes PKA phosphorylation [39, 49]. The increased PKA phosphorylation in DHA-fed pigs might be expected to promote lipolysis. However, the effects of PKA are multifarious and increased activation of PKA may be important for the activation of proteins and transcription factors in lipid metabolism regulation. The overall effect of DHA in this study was an increase in cellular and tissue TAG and adipocyte size. In support of this, there is evidence that lipogenic genes such as ADRP and DGAT1 are up-regulated by PKA activation [50, 51]. We found that the expression of lipogenic genes, ADRP and DGAT was elevated in adipose tissue and primary adipocytes by DHA. Adipose differentiation related protein (ADRP) is a 50-kDa protein, which facilitates long-chain polyunsaturated fatty acid (LCPUFA) uptake and storage, supports stabilization of lipid droplets [50] and is strongly induced in cells with increased lipid load [51]. In the current study, ADRP expression increased along with TAG content and adipocyte size in pigs fed DHA. The expression of ADRP is induced by dibutylyl cAMP and blocked by a PKA inhibitor in human hepatoma cells [52]. The other lipogenesis related protein, diglyceride acyltransferase 1 (DGAT1) is expressed in most tissues, especially those that make large amounts of TAG, including liver, adipose tissue and mammary gland. Adipose tissue has the highest levels of mRNA for DGAT 1 among all tissues [53]. Hamster fibroblasts incubated with cell-permeable cAMP analogues have increased TAG synthesis, which is attributed to increased DGAT activity [54]. Furthermore, DGAT1 has two consensus PKA phosphorylation sites (T15 and S244) [53, 55]. Therefore, it is conceivable that increased activation of PKA is partly responsible for the elevated expression of ADRP and DGAT in pig adipose tissue and adipocytes exposed to DHA.

Long chain n-3 fatty acids are effective in reducing plasma TAG, which in turn may suppress adipose tissue inflammation and enhance cardiac, hepatic and skeletal muscle fatty acid *β*-oxidation contributing to reduced FFA delivery to the liver [18, 56]. ACOX1 and PPAR*α* mRNA are increased by 300 to 9400 mg of dietary DHA in finishing pigs [19]. In our experiment, expression of ACOX1 mRNA in subcutaneous fat was increased in both BT- and DHA- fed pigs, but expression of the mitochondria *β*-oxidation gene, CPT1*α* was only increased in the DHA-fed pigs (Fig. 4c). The data suggest that fatty acid oxidation may be increased in porcine adipose tissue by DHA. Thus, increased adipose tissue fatty acid oxidation may contribute to the lowered serum TAG in DHA fed pigs.

The mechanisms by which dietary n-3 PUFA suppress hepatic lipogenesis and TAG secretion and induce fatty acid oxidation are well-known [57–59]. These mechanisms have also been confirmed in our previous pig studies [17, 18, 23]. Our laboratory extended these mechanisms to show that DHA down-regulates forkhead box O (FoxO) target genes, such as microsomal triacylglycerol transfer protein and apolipoprotein C3 to inhibit VLDL-TG assembly and synthesis [23]. In the current study, using the same experimental setting, we focused on the adipose tissue-specific effects of DHA. This is based on the different roles of the adipose and liver in metabolic regulation. Combining our previous results in which we elucidated mechanisms of the DHA effects on the liver [23] and the present study, it is very clear that, in addition to inhibition of hepatic TAG synthesis, DHA promoted TAG accumulation in the adipose tissue. This conclusion is supported by a similar study in which DHA promotes adipogenesis [35].

## Conclusion

Based on our observation, the adipocytes of DHA fed pigs had relatively larger adipocyte size and more TAG accumulation compared with SBO-fed group. DHA is known as a PPARγ ligand, thus it is similar to the TZD family of drugs (PPARγ agonists), and our data confirm the gene expression results of Ranganathan et al. [60]. In addition, dietary DHA in growing pigs reduced plasma concentration of TAG. This effect was partly explained by the effect of DHA to promote trapping TAG into adipocytes through up-regulation of ADRP and DGAT1. Supplementation with DHA also increased expression of genes involved in adipocyte fatty acid oxidation (ACOX1 and CPT1α). Therefore, our results suggest a direct effect of DHA on adipocyte metabolism, resulting in an improved overall metabolic profile.

## Additional file

Additional file 1: Supplementary data: Methods. **Figure S1.** (a) The retention time of PUFA NO2. Mix standard, animal source (Sigma-Aldrich). **Table S1.** Fatty acid compositions of the dietary oils (%, total fatty acids). **Table S2.** Fatty acid compositions of the experimental diets (%, total fatty acids). **Table S3.** Fatty acid of plasma total lipids in different dietary treatments (fatty acid content, μmol / L and percent (%)). **Table S4.** Fatty acid compositions of adipose tissue total lipids in different dietary treatments (fatty acid content, μmol /g tissue and percent (%)). **Table S5.** The response factors for flame ionization detector with each methyl fatty acids. (DOCX 177 kb)

## Abbreviations

ACOX1: Acyl-coenzymes A oxidase 1
ADRP: Adipose differentiated related protein
aP2: Adipocyte protein 2
ATGL: Adipose triglyceride lipase
C/EBPα: CCAAT-enhancer binding protein α
CGI58: Comparative gene identifcation 58
CPT1α: Carnitine palmitoyltransferase 1 α
DGAT1: Diacylglycerol acyltransferase 1
DHA: Docosahexaenoic acid
FA: Fatty acid
FBS: Fetal bovine serum
H&E: Hematoxylin-eosin
HSL: Hormone sensitive lipase
LA: Linoleic acid
LPL: Lipoprotein lipase
MRI: Magnetic resonance imaging
OA: Oleic acid
PKA: Protein kinase A
PPARα: Proliferator-activated receptors α
PPARγ: Proliferator-activated receptor γ
PUFA: Polyunsaturated fatty acid
S/V cell: Stromal/vascular cell
SAA: Serum amyloid A
SBO: Soybean oil
SCAT: Subcutaneous adipose tissue
SREBP-1: Sterol regulatory element binding protein
TAG: Triacylglycerol
