# Supplementary material for: Docosahexaenoic acid increases accumulation of adipocyte triacylglycerol through up-regulation of lipogenic gene expression in pigs
Source: Lipids Health Dis. 2017 Feb 7;16:33. doi: 10.1186/s12944-017-0428-3 (PMC5297193; doi:10.1186/s12944-017-0428-3)

***Supplementary data***

Methods

The lipids in feed, plasma and adipose tissues were extracted according to the procedure of Folch et al (1957) ^(1)^, with heptadecanoic acid (Sigma-Aldrich, St. Louis, MO, USA) added to each sample as an internal standard before extraction. We used PUFA NO. 2 Mix (Sigma, St. Louis, MO) as our external standard fatty acid methyl ester (FAME), which was analyzed directly by GC.

Total extracted lipids were converted to fatty acid methyl esters and separated by gas chromatography, using the modified procedure of Lee et al. ^(2)^. In brief, total lipids were converted to fatty acid methyl esters (FAME) and separated and quantified using a gas chromatograph (GC, Varian Star 3400CX in Varian Associate, Inc., California, USA) equipped with a flame ionization detector (FID) and fitted with a Varian ® CPWax-58 CB WCOT Fused Silica capillary column (30m X 0.53 mm, DF=1.0 μM). The injection volume was 0.1 μL and the mode was split-less direct injection. The oven temperature was 240°C and the detector temperature was 260°C. Peaks were identified by retention times relative to individual FAME standards. The amount of an individual fatty acid was calculated based on the internal standard ^(3,4)^. Response factors for each FAME were indicated (Table S4). SBO: soybean oil, DHA: docosahexaenoic acid oil and BT: beef tallow and ND indicated not detected.

***Reference***

1. Folch, J.; Lees, M.; Sloane-Stanley, G., A simple method for the isolation and purification of total lipids from animal tissues. J Biol Chem 1957, *226* (1), 497-509.

2. Lee, W. J.; Lee, M. H.; Su, N. W., Characteristics of papaya seed oils obtained by extrusion–expelling processes. J Sci Food Agric 2011; *91* (13), 2348-2354.

3. Glaser, C.; Demmelmair, H.; Koletzko, B., High-throughput analysis of total plasma fatty acid composition with direct in situ transesterification. PLoS One 2010; *5* (8), e12045.

4. Masood, A.; Stark, K. D.; Salem, N., A simplified and efficient method for the analysis of fatty acid methyl esters suitable for large clinical studies. J Lipid Res 2005; *46* (10), 2299-2305.

Figure S1. (a) The retention time of PUFA NO2. Mix standard, animal source (Sigma-Aldrich).


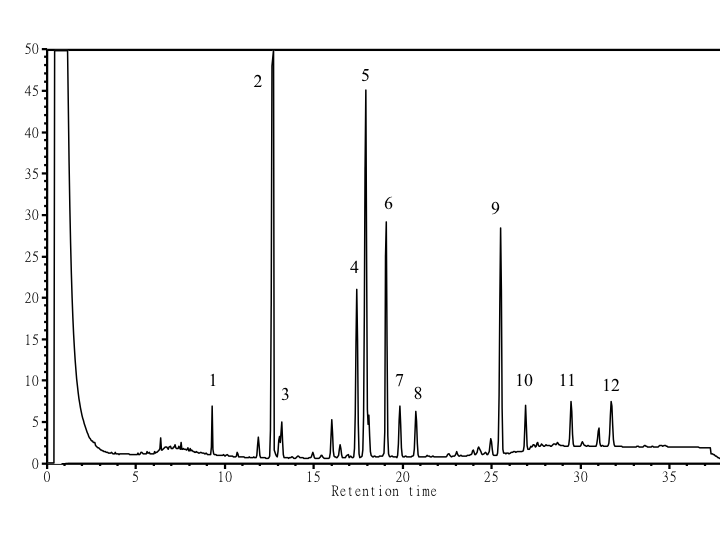


Elution order: 1=C14:0, 2= C16:0, 3= C16:1n7, 4=C18:0, 5=C18:1n9, 6= C18:2n6, 7= C18:3n6, 8= C18:3n3, 9= C20 :4n6, 10= C20:5n3, 11= C22:4n6, 12= C22:6n3.

Table S1. Fatty acid compositions of the dietary oils (%, total fatty acids).

| Diet^1^ | SBO | | DHA | | BT | |  |
| --- | --- | --- | --- | --- | --- | --- | --- |
| Fatty acid^2^ | |  | |  | |  | |
| C14:0 | | 0.11 ± 0.01^b^ | | 16.97 ± 4.93 ^a^ | | 2.59 ± 0.14 ^b^ | |
| C16:0 | | 11.50 ± 0.53 ^b^ | | 44.51 ± 9.6 ^a^ | | 32.20 ± 1.30 ^a^ | |
| C16:1n7 | | 0.10 ± 0.00 ^b^ | | 0.40 ± 0.08 ^b^ | | 1.52 ± 0.09 ^a^ | |
| C18:0 | | 4.21 ± 0.15 ^a^ | | 2.43 ± 0.51 ^b^ | | 15.80 ± 0.53 ^a^ | |
| C18:1n9 | | 23.25 ± 0.50 ^b^ | | 17.09 ± 3.10 ^b^ | | 36.43 ±1.19 ^a^ | |
| C18:2n6 | | 47.80 ± 0.74 ^a^ | | 3.20 ± 0.47 ^b^ | | 5.19 ± 0.09 ^b^ | |
| C18:3n6 | | 5.47 ± 0.01 ^a^ | | 0.27 ± 0.11 ^ns^ | | 0.31 ± 0.03 ^ns^ | |
| C18:3n3 | | 3.31±0.28^b^ | | 1.11±0.45^b^ | | 0.80±0.14^b^ | |
| C20:4n6 | | 4.87 ± 3.57* | | ND | | ND | |
| C20:5n3 | | ND | | 0.67 ± 0.09 * | | ND | |
| C22:4n6 | | ND | | 1.65 ± 0.37 * | | ND | |
| C22:6n3 | | ND | | 1.69 ± 0.35 * | | ND | |
| ∑ Saturates | | 15.82 ± 0.23 ^c^ | | 63.91 ± 5.01^a^ | | 50.59 ± 0.66 ^b^ | |
| ∑ Monounsaturates | | 23.35 ± 0.25 ^b^ | | 17.49 ± 1.59 ^c^ | | 37.95 ± 0.64^a^ | |
| ∑ n-3 PUFA | | 0.46 ± 0.06 ^b^ | | 2.63 ± 0.18 ^a^ | | 0.31 ± 0.01 ^b^ | |
| ∑ n-6 PUFA | | 53.13 ± 0.31 ^a^ | | 14.36 ± 0.97 ^b^ | | 10.38 ± 0.04^c^ | |

1. Oil were SBO=soybean oil, DHA= docosahexaenoic acid oil and BT= beef tallow. ND indicated not detected.
2. One way ANOVA followed by Tukey's post hoc test was performed for multiple comparisons. Means in a row with different superscripts were significantly different (p ≤ 0.05). If there is ND in one of the treatments, we used a t test to compare the two groups. *Values were significantly different (p ≤ 0.05) as determined by Student’s t test. ns, indicated no significant differences were observed between the groups. Values were mean±SD, n=3.

Fatty acid partial sums of each diet composition. ∑ Saturates = C14:0 + C16:0+C18:0, ∑ Monounsaturates= C16:1n7+C18:1n9, ∑ n-3 PUFA= C18:3n3+C20:5n3+C22:6n3 and ∑ n-6 PUFA= C18:2n6+C18:3n6+C20:4n6+C22:4n6.

Table S2. Fatty acid compositions of the experimental diets (%, total fatty acids).

| Diet^1^ | SBO | | DHA | | BT | |  |
| --- | --- | --- | --- | --- | --- | --- | --- |
| Fatty acid^2^ | |  | |  | |  | |
| C14:0 | | 3.89 ± 0.71 ^ns^ | | 4.71 ± 0.13 ^ns^ | | 4.95 ± 1.32 ^ns^ | |
| C16:0 | | 19.17 ± 2.25 ^ns^ | | 16.96 ± 1.6 ^ns^ | | 23.76 ± 7.06 ^ns^ | |
| C16:1n7 | | 0.94 ± 0.07 ^ns^ | | 1.64 ± 0.24 ^ns^ | | 1.44 ± 0.47 ^ns^ | |
| C18:0 | | 8.03 ± 2.17 ^a^ | | 4.19 ± 0.17 ^b^ | | 9.18 ± 0.49 ^a^ | |
| C18:1n9 | | 19.70 ± 2.19 ^ns^ | | 26.88 ± 4.81 ^ns^ | | 27.00 ± 1.45 ^ns^ | |
| C18:2n6 | | 43.82 ± 2.41 ^a^ | | 34.64 ± 1.61 ^b^ | | 32.88 ± 2.29 ^b^ | |
| C18:3n6 | | 0.10 ± 0.02 ^b^ | | 0.27 ± 0.02 ^a^ | | ND | |
| C18:3n3 | | 3.31 ± 0.28 ^b^ | | 1.11 ± 0.45 ^b^ | | 0.80 ± 0.14 ^b^ | |
| C20:4n6 | | 0.94 ± 0.07 * | | ND | | ND | |
| C20:5n3 | | 0.10 ± 0.08 | | 0.26 ± 0.03 ^a^ | | ND | |
| C22:4n6 | | ND | | 0.13 ± 0.03 * | | ND | |
| C22:6n3 | | ND | | 9.22 ± 0.04 * | | ND | |
| ∑ Saturates | | 31.09 ± 1.71 ^b^ | | 25.85 ± 0.63 ^c^ | | 37.98 ± 2.95 ^a^ | |
| ∑ Monounsaturates | | 20.63 ± 1.13 ^b^ | | 28.52 ± 2.53 ^a^ | | 28.44 ± 0.96 ^a^ | |
| ∑ n-3 PUFA | | 3.41 ± 0.12 ^b^ | | 10.59 ± 0.41 ^a^ | | 0.80 ± 0.05 ^c^ | |
| ∑ n-6 PUFA | | 44.86 ± 0.7 ^a^ | | 35.03 ± 0.42 ^b^ | | 32.88 ± 0.57 ^c^ | |

1. Diets were SBO=soybean oil, DHA= docosahexaenoic acid oil and BT= beef tallow. ND indicated not detected.
2. One way ANOVA followed by Tukey's post hoc test was performed for multiple comparisons. Means in a row with different superscripts were significantly different (p ≤ 0.05). If there is ND in one of the treatments, we used a t test to compare the two groups. *Values were significantly different (p ≤ 0.05) as determined by Student’s t test. ns, indicated no significant differences were observed between the groups. Values were mean±SD, n=3.

Fatty acid partial sums of each diet composition. ∑ Saturates = C14:0 + C16:0+C18:0, ∑ Monounsaturates= C16:1n7+C18:1n9, ∑ n-3 PUFA= C18:3n3+C20:5n3+C22:6n3 and ∑ n-6 PUFA= C18:2n6+C18:3n6+C20:4n6+C22:4n6.

Table S3. Fatty acid of plasma total lipids in different dietary treatments (fatty acid content, μmol / L and percent (%)).

| Plasma | | | | | | | | | | | | | | |  |
| --- | --- | --- | --- | --- | --- | --- | --- | --- | --- | --- | --- | --- | --- | --- | --- |
| Diet^1^ | SBO | |  | | DHA | | |  | | | BT | | |  | |
|  | | FA concentration | | FA composition (%) | | FA concentration | | | FA composition (%) | | | FA concentration | FA composition (%) | | |
| Fatty acid^2^ | |  | |  | |  | | |  | | |  |  | | |
| C14:0 | | 23.19 ± 0.01^ns^ | | 3.5 ± 0.0 | | 23.91 ± 2.86^ns^ | | | 3.5 ± 0.4 | | | 27.92 ± 3.14^ns^ | 4.4 ± 0.5 | | |
| C16:0 | | 138.76 ± 11.53^ns^ | | 20.9 ± 1.7 | | 164.28 ± 29.98^ns^ | | | 24.3 ± 4.4 | | | 186.61 ± 4.09^ns^ | 29.6 ± 0.7 | | |
| C16:1n7 | | 38.58 ± 7.59^ns^ | | 5.8 ± 1.1 | | 27.64 ± 1.01^ns^ | | | 4.1 ± 0.2 | | | 47.29 ± 9.21^ns^ | 7.5 ± 1.5 | | |
| C18:0 | | 110.18 ± 14.90^ns^ | | 16.6 ± 2.2 | | 82.67 ± 12.64^ns^ | | | 12.2 ± 1.9 | | | 108.76 ± 7.23^ns^ | 17.3 ± 1.2 | | |
| C18:1n9 | | 141.11 ± 26.75^ns^ | | 21.3 ± 4.0 | | 167.47 ± 49.95^ab^ | | | 24.8 ± 7.4 | | | 169.13 ± 14.20^ns^ | 26.8 ± 2.3 | | |
| C18:2n6 | | 173.22 ± 30.12^a^ | | 26.1 ± 4.5 | | 112.08 ± 26.47^ns^ | | | 16.6 ± 3.9 | | | 78.64 ± 4.48^b^ | 12.5 ± 0.7 | | |
| C18:3n6 | | 13.51 ± 9.02^ns^ | | 2.0 ± 1.4 | | 20.45 ± 15.19^ns^ | | | 3.0 ± 0.2 | | | 2.73 ± 0.87^ns^ | 0.4 ± 0.1 | | |
| C18:3n3 | | 6.15 ± 1.51^b^ | | 0.9 ± 0.2 | | 18.29 ± 2.83^a^ | | | 2.7 ± 0.4 | | | 9.52 ± 3.08^b^ | 1.5 ± 0.5 | | |
| C20:4n6 | | 16.55 ± 3.55* | | 2.5 ± 0.5 | | 11.96 ± 2.90 | | | 1.8 ± 0.4 | | | ND | ND | | |
| C20:5n3 | | 2.83 ± 0.44 | | 0.4 ± 0.1 | | 20.14 ± 1.94* | | | 3.0  ± 0.3 | | | ND | ND | | |
| C22:4n6 | | ND | | ND | | ND | | | ND | | | ND | ND | | |
| C22:6n3 | | ND | | ND | | 26.33 ± 2.25 | | | 3.9 ± 0.3 | | | ND | ND | | |
| Fatty acid partial sums | | | | | | | | | | | | | | |  |
| ∑ Saturates | | 272.13 ± 8.81^b^ | | 41.0 ± 1.3 | | | 270.87 ±15.16^b^ | | | 40.1 ± 2.2 | | 323.29 ± 4.82^a^ | 51.3 ± 0.8 | | |
| ∑ Monounsaturates | | 179.69 ± 17.17^b^ | | 27.1 ± 2.6 | | | 195.11 ± 25.48^ab^ | | | 28.9 ± 3.8 | | 216.42 ±11.70^a^ | 34.3 ± 1.9 | | |
| ∑ n-3 PUFA | | 8.98 ± 0.65^b^ | | 1.4 ± 0.1 | | | 64.76 ± 2.34^a^ | | | 9.6 ± 0.4 | | 9.52 ± 1.03^b^ | 1.5 ± 0.2 | | |
| ∑ n-6 PUFA | | 203.28 ± 10.68^a^ | | 30.6 ± 1.4 | | | 144.49 ± 7.72^b^ | | | 21.4 ± 1.1 | | 81.37 ± 1.34^c^ | 12.9 ± 0.2 | | |

Diets were SBO=soybean oil, DHA= docosahexaenoic acid oil and BT= beef tallow. ND indicated not detected.

One way ANOVA followed by Tukey's post hoc test was performed for multiple comparisons. Means in a row with different superscripts were significantly different (p ≤0.05). If there is ND in one the treatment, we used t test to compare with two groups. *Values were significantly different (p ≤0.05) as determined by Student’s t test. ns, indicated no significant differences were observed between the groups. Values were mean±SD, n=6.

Fatty acid partial sums of each plasma composition. Fatty acid content, μmol/L. ∑ Saturates = C14:0 + C16:0+C18:0, ∑ Monounsaturates= C16:1n7+C18:1n9, ∑ n-3 PUFA= C18:3n3+C20:5n3+C22:6n3 and ∑ n-6 PUFA= C18:2n6+C18:3n6+C20:4n6+C22:4n6.

Plama from SBO-fed pigs had a high concentration of C18:2n-6, DHA-fed pigs had considerable C20:5n-3 and C22:6n-3, and BT-fed pigs had a high concentration of the saturated fatty acids and monounsaturated fatty acids. The fatty acid compositions in plamsa were as expected from the diet compositions.

Table S4. Fatty acid compositions of adipose tissue total lipids in different dietary treatments (fatty acid content, μmol /g tissue and percent (%)).

| Adipose tissue | | | | | | | | | | | |  |
| --- | --- | --- | --- | --- | --- | --- | --- | --- | --- | --- | --- | --- |
| Diet^1^ | SBO | |  | | DHA | |  | | BT |  | |  |
|  | | FA concentration | | FA composition (%) | | FA concentration | | FA composition (%) | FA concentration | | FA composition (%) | |
| Fatty acid^2^ | |  | |  | |  | |  |  | |  | |
| C14:0 | | 27.91 ± 8.93^ns^ | | 3.7±1.8 | | 107.05±45.15^ns^ | | 3.3 ± 1.4 | 83.51 ± 40.68^ns^ | | 3.0±1.5 | |
| C16:0 | | 200.80 ± 7.68^b^ | | 26.5 ± 1.5 | | 924.54 ± 216.82^a^ | | 28.4 ± 6.7 | 750.25 ± 49.35^a^ | | 27.0 ± 12.6 | |
| C16:1n7 | | 31.22 ± 4.20^b^ | | 4.1 ± 0.8 | | 139.11 ± 12.68^a^ | | 4.3 ± 0.4 | 133.19 ± 15.95^a^ | | 4.8 ± 2.4 | |
| C18:0 | | 71.46 ± 7.35^b^ | | 9.4 ± 1.5 | | 321.46 ± 23.93^a^ | | 9.9 ± 0.7 | 255.39 ± 95.07^a^ | | 9.2 ± 3.4 | |
| C18:1n9 | | 182.71 ± 10.74^b^ | | 24.1 ± 2.1 | | 935.14 ± 47.23^a^ | | 28.7 ±1.5 | 990.35 ± 5.20^a^ | | 35.7 ± 18.2 | |
| C18:2n6 | | 220.51 ± 7.97^b^ | | 29.1 ± 1.6 | | 647.73 ± 3.66^a^ | | 19.9 ± 0.1 | 529.27 ± 101.84^a^ | | 19.1 ± 9.1 | |
| C18:3n6 | | ND | | ND | | 2.28 ± 0.12 | | 0.1 ± 0.0 | ND | | ND | |
| C18:3n3 | | 10.51 ± 1.67^b^ | | 1.4 ± 0.3 | | 38.71 ± 5.31^a^ | | 1.2 ± 0.2 | 33.00 ± 5.51^a^ | | 1.2 ± 0.6 | |
| C20:4n6 | | 4.24 ± 0.14 | | 0.6 ± 0.0 | | 10.76 ± 1.98* | | 0.3 ± 0.1 | ND | | ND | |
| C20:5n3 | | ND | | ND | | 13.52 ± 3.88^ns^ | | 0.4 ± 0.1 | ND | | ND | |
| C22:4n6 | | 2.92 ± 0.06 | | 0.4 ± 0.0 | | 32.84 ± 3.33* | | 1.0 ± 0.1 | ND | | ND | |
| C22:6n3 | | 227.68 ± 2.04 | | 0.7 ± 0.1 | | 84.62 ± 10.84* | | 2.6 ± 0.3 | ND | | ND | |
| Fatty acid partial sums | | | | | | | | | | | |  |
| ∑ Saturates | | 300.17 ± 7.99^c^ | | 40.0 ± 1.6 | | 1353.05 ± 95.30^a^ | | 41.5 ± 2.9 | 1089.16 ± 61.70^b^ | | 39.3 ± 5.8 |  |
| ∑ Monounsaturates | | 213.93 ± 7.47^b^ | | 28.3 ± 1.5 | | 1074.25 ± 29.96^b^ | | 33.0 ± 0.9 | 1123.55 ± 10.57^a^ | | 4.05 ± 10.1 |  |
| ∑ n-3 PUFA | | 15.41 ± 0.66^b^ | | 2.0 ± 0.1 | | 136.85 ± 2.34^a^ | | 4.2 ± 0.2 | 33.00 ± 1.84^b^ | | 1.2 ± 0.2 |  |
| ∑ n-6 PUFA | | 227.68 ± 2.04^a^ | | 30.1 ± 0.4 | | 693.60 ± 2.27^a^ | | 21.3 ± 0.1 | 529.27 ± 25.46^b^ | | 19.1 ± 2.3 |  |

Diets were SBO=soybean oil, DHA= docosahexaenoic acid oil and BT= beef tallow. ND indicated not detected.

One way ANOVA followed by Tukey's post hoc test was performed for multiple comparisons. Means in a row with different superscripts were significantly different (p ≤0.05). If there is ND in one the treatment, we used t test to compare with two groups. *Values were significantly different (p ≤0.05) as determined by Student’s t test. ns, indicated no significant differences were observed between the groups. Values were mean± SD, n=6.

Fatty acid partial sums of each plasma composition. Fatty acid content, μmol/L. ∑ Saturates = C14:0 + C16:0+C18:0, ∑ Monounsaturates= C16:1n7+C18:1n9, ∑ n-3 PUFA= C18:3n3+C20:5n3+C22:6n3 and ∑ n-6 PUFA= C18:2n6+C18:3n6+C20:4n6+C22:4n6.

Adipose tissue from SBO-fed pigs accumulated considerable C18:2n6, whereas that from DHA-fed pigs accumulated C20:5n3 and C22:6n3, and BT-fed pigs accumulated considerable saturated and monounsaturated but much less polyunsaturated fatty acids. All of these results were as expected based on the diet compositions and plasma fatty acids. It should be noted that the high concentration of C16:0, C18:0 and C18:1 represent both diet input and endogenous synthesis.

Table S5. The response factors for flame ionization detector with each methyl fatty acids.


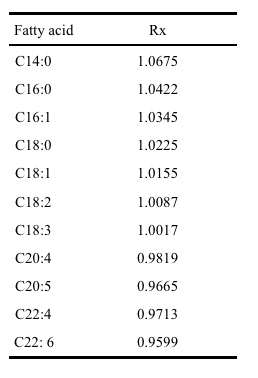

Supplement: Additional file 1: — Supplementary data: Methods. Figure S1. (a) The retention time of PUFA NO2. Mix standard, animal source (Sigma-Aldrich). Table S1. Fatty acid compositions of the dietary oils (%, total fatty acids). Table S2. Fatty acid compositions of the experimental diets (%, total fatty acids). Table S3. Fatty acid of plasma total lipids in different dietary treatments (fatty acid content, μmol / L and percent (%)). Table S4. Fatty acid compositions of adipose tissue total lipids in different dietary treatments (fatty acid content, μmol /g tissue and percent (%)). Table S5. The response factors for flame ionization detector with each methyl fatty acids. (DOCX 177 kb) [file 12944_2017_428_MOESM1_ESM.docx]
